# The role of RANK ligand/OPG system in bone erosions in rheumatoid arthritis

**DOI:** 10.1186/ar3720

**Published:** 2012-03-08

**Authors:** Piet Geusens

**Affiliations:** 1 Department of Internal Medicine, Subdivision of Rheumatology, Maastricht University Medical Center, Maastricht, The Netherlands; 2 Biomedical Research Institute, University Hasselt, Belgium E-mail: piet.geusens@scarlet.be

## 

There is still growing insight on the links between the immune system and bone at the anatomical, vascular, cellular and molecular level. At the anatomical level, bone is at the inside in direct contact with the bone marrow and at the outside with structures that are involved in chronic inflammatory rheumatic diseases (entheses, periosteum, calcified cartilage). At the cellular and molecular level, many bilateral cross-talks between the immune system and bone have been described, in normal physiological conditions and during inflammation. In addition, in the presence of bone erosions at the sites of inflammation, direct contact is available between the bone marrow and the joint cavity.

Bone resorption is increased in RA. This has been demonstrated by the presence of activated osteoclasts in the pannus at the site of bone erosions and in subchondral osteitis.

The final cellular pathway of the attack of chronic or recurrent inflammation on bone is the recruitment and activation of osteoclasts. One of the central molecular pathways in this process is RANK ligand/OPG. In normal conditions, the osteoblast is the main regulator of bone resorption by this pathway. In rheumatoid arthritis, RANK ligand is also produced by inflammatory cells, including activated T- and B-cells, which are present in synovitis and in subchondral osteitis. By playing a central role in erosion formation, the osteoclast is not only responsible for functional handicap resulting from bone destruction at long term, but also for allowing the joint space to have direct access with subchondral bone marrow and its vast reserve of stem cells and B-cells.

Inhibition of osteoclasts by denosumab, a humanized antibody that selectively binds RANK ligand, has revealed that the occurrence of erosions and peri-articular bone loss can be halted, however without affecting synovial inflammation. This disconnect between inflammation and bone destruction opens new ways to separately focus treatment on inflammation and osteoclastogenesis for preventing and/or minimizing the connection between joints and subchondral bone and bone marrow.

